# Attributable patient risk in nuclear medicine procedures and establishment of diagnostic reference levels

**DOI:** 10.1002/acm2.13658

**Published:** 2022-12-28

**Authors:** Julio Cesar de Souza Ribeiro, Janaína Dutra Silvestre de Mendes, Lidia V. de Sá

**Affiliations:** ^1^ Instituto Nacional de Câncer (INCA) Rio de Janeiro Brazil; ^2^ Instituto de Radioproteção e Dosimetria (DIFME/IRD/CNEN) Rio de Janeiro Brazil

**Keywords:** absorbed dose, administered activity, diagnostic reference levels, nuclear medicine

## Abstract

The assessment of risk related to medical exposures as a justification tool to assist decision‐making of the medical team is not available in clinical routine. The determination of diagnostic reference levels (DRLs) for nuclear medicine (NM) procedures has been proposed as an optimization tool, but this tool has still been aimed at a standard adult individual. It is known that the activity administered, and the consequent absorbed doses in critical organs, represents the risk of a procedure being cancer induction the greatest concern, especially for young patients. Thus, the adjustment of administered activity and procedure risk to promote risk–benefit assessment is a promising tool for routine clinical use. This work aims to present a tool for determining DRLs in the administered activity related to the patient's characteristics—age group, sex, and body mass index (BMI), in order to assist the medical decision regarding the risk–benefit ratio. Thus, it is possible to assess the risk of carcinogenesis in groups of patients, considering absorbed doses in organs, cancer incidence, and mortality rates in our country. NIREA is an IT tool developed in PHP language for web environment as a friendly software. It allows the establishment of DRL and risk of cancer induced by radiation assessment through the estimation of absorbed doses in specific organs and based on the risk methodology of BEIR VII. The absorbed and effective doses were estimated based on the dose conversion factors of the radiopharmaceuticals published by the International Commission on Radiological Protection adjusted for the patient groups. Based on data from 2256 patients who underwent diagnostic procedures at National Cancer Institute between 2017 and 2019, the program was used, resulting in important information for conducting the clinical routine extracted as DRL, absorbed doses, and risk assessments, considering patient‐specific data such as age, sex, and BMI. The methodology developed in this work allows NM services to keep their data available and updated regarding local DRLs, in addition to allowing the nuclear physician to know the risk of each procedure performed, extracted by individual characteristics of the patient. The affirmative is significant because the data could be used by the regulatory body of practices with ionizing radiation in Brazil to establish a reference level in Activity that has not yet existed in the country.

## 1 INTRODUCTION

Ionizing radiation has been widely used in different segments of society, mainly in scientific research and medical applications. Many studies were developed aiming at the possible implications of the use of this technology and the associated risks, whose analysis led the International Commission on Radiological Protection (ICRP) to introduce the requirement of justification for its use in patients since 1955.^1^ In 1996, the adoption of diagnostic reference levels (DRLs) for procedures in the nuclear medicine (NM) has been recommended, in accordance with the practices adopted and the legislation in force in each country or region. It is known that ICRP has emphasized the importance of accurately determining the average dose levels, or administered activity, received by patients in each medical procedure. However, bibliographic references that address the need to know and even the advantages that the determination of the doses involved in each procedure are scarce, or rather limited, which may favor the non‐standardization of techniques, and possible increase in associated radiological risks. Therefore, it is extremely important to evaluate and estimate the doses received by the population, in order to ensure that the risk associated is inferior to the benefits thus guaranteeing the principle of justification. In European Community (EC) to assess the exposure situation in a medical field, the Dose DataMed I project was developed in 2004 and, later in 2011, the Dose DataMed II was carried out as a major survey in member countries. The results, published in Radiation Protection 154 Report,^2^ defined methodologies to address collective doses. Directive 2013/59/EURATOM highlights some important technological and scientific developments that have led to a notable increase in patient exposure levels. It also reinforces the close attention to justification of the medical radiological exposures, including asymptomatic persons. The document strengthens the requirements regarding the information to be provided to patients, the recording and reporting of doses resulting from medical procedures to the use of DRLs, and the availability of dose indicating tools. The EC Member States shall ensure the establishment, periodic review, and use of DRLs for radiological examinations taking into account, when available, the DRLs recommended at the European level. In Brazil, national legislation^3^, ^4^ also presents requirements for DRLs and even the need for control and analysis of doses received in medical exposures; however, this requirement has not been fully met. Some information on this subject can be obtained, either through the Ministry of Health, or the National Nuclear Energy Commission (CNEN), regulatory bodies of the medical practices involving ionizing radiation. No references are found for NM, which may favor non‐standardization of techniques, uncontrolled exposures, and possibly increasing radiological risks associated with these procedures. Besides, the doses in NM are directly related to patient's weight, in which many protocols and guidelines define the activity administered by the patient's body mass (Bq/kg), so this body index mass should be considered.

Another important focus of international efforts would be the assistance to survey carried out by UNSCEAR (United Nations Scientific Committee on the Effects of Atomic Radiation) where Member States should provide national data on the types and number of exams and treatments performed in each country, estimating the doses involved to obtain a world map of current medical exposures.^5^

Regarding the risk analysis resulting from exposure, especially the risk associated with radioinduced cancer, most international organizations, based on theoretical and experimental evidence and mainly with a view to radiological protection, postulate that minimal exposure to radiation may be sufficient to promote cell alterations, increasing the probability of cancer induction.^6^, ^7^ A radioinduced cancer risk model defines the relationship between the radiation absorbed dose in a critical organ and the subsequent risk of mortality or morbidity resulting of this exposure. To address these concerns and also provide a tool for practical use in clinical routine, a methodology was proposed that could model the consequences arising from the use of radiation in NM patients with the aim of delimiting the administration of potentially excessive activities, an estimate of the effective dose received by groups of patients for each type of exam, making it possible to identify the risk, discuss the effect of the response time on the expected detriment and also support the decision‐making process of the medical staff. The methodology adopted for calculating the probability of induction and risk of developing radioinduced cancer was provided by the BEIR VII report.^7^ The mathematical model was used to estimate the lifetime attributable risk (LAR) that was calculated by patient categories such as gender, age, and body mass index (BMI). The use of a risk analysis for NM procedures can contribute considerably to the control and standardization of the technique, offering the medical community tools to perform exams and highly complex therapies considering the risks involved.

## 2 RISK MODEL

The BEIR VII report^7^ presents risk factors and models to be used primarily in estimating the risk of carcinogenesis for low doses of radiation. Two risk calculation models are used: the excess relative risk (ERR) and the excess absolute risk (EAR). These models allow the calculation of cancer risk at a given time after exposure, and its value depends on the age and sex of the individual at the time of exposure.

The ERR model assumes that the cancer incidence or mortality rate in the radiation‐exposed population depends on the basal rate in the general population. The EAR model assumes the hypothesis that the incidence or mortality rate in the population exposed to radiation is independent of the rate in the general population.

The mathematical model adopted by the BEIR VII Committee to estimate the ERR or EAR for solid tumors (except breast, thyroid, and nonmelanoma cancer) is given by the following equation:

(1) $$\text{ERR}\;\text{and}\;\text{EAR}=\beta_{s}D\;\exp\left(\gamma e^{*}\right){\left(\frac{a}{60}\right)}^{\eta }$$

where *D* is the equivalent dose (Sv), *β_s_*, γ, and *η* are specific parameters of the ERR and EAR for various organs and for each sex; *e* is the age at exposure (in years), and *e* ^*^ = (*e* − 30)/10 for *e* < 30 years and 0 for *e* ≥ 30 years; *a* is the age reached.

Ideally, risk models would be developed from data collected from randomly selected individuals from a population for which risk estimation is desired. However, data from specific populations of interest are hardly available in sufficient quantity or exposure level to allow adequate statistical modeling. Thus, risk models are often developed using data from one population (usually not even a random sample) with the aim of estimating risks in other populations. Interpopulation extrapolations of this type are referred to as “transport” of the model from one population to another, which must be done carefully, especially considering the epidemiological characteristics of the population to which the model will be applied.^7^

The LAR is the difference between the rate of a disease between the exposed population and the unexposed population, which is an estimate of the probability of developing premature cancer due to exposure to radiation throughout the individual's lifetime. The LAR depends on the age of the individual at the time of exposure and incorporates several additional factors, such as the latency period between exposure and the time when cancer arises, as well as the dose‐effectiveness factor and dose rate (DDREF, dose and dose‐rate effectiveness factor). DDREF is defined as the factor by which radiation cancer risk is observed after a high dose and dose rate, which should be reduced when radiation is deposited at low dose rates or in a series of small, fractionated doses. The BEIR VII Committee sets the value of 1.5 for the DDREF.^7^

The BEIR VII Committee uses LAR to transport the cancer risk estimation model from one population to another. For an individual exposed to a dose *D* at an age *e*, the LAR is given by the following equation:

(2) $$\mathrm{LAR}\left(D,e\right)=\sum_{e+L}^{100}M\left(D,e,a\right)\cdot \frac{S\left(a\right)}{S\left(e\right)}$$

The sum in the previous equation is performed in the interval between *a* = *e* + *L* and *a* = 100 years, where *e* is the age at exposure, and *L* is the latency period (2 years for leukemia and 5 years for solid tumors); *a* is the age reached. *S*(*a*) is the probability of surviving to age *a*, and *S*(*a*)/*S*(*e*) is the probability of surviving to age *a* conditional on the probability of surviving to age *e*.

The term *M*(*D*,*e*,*a*) can be calculated using ERR or EAR model. Using the ERR model to estimate the risk of cancer incidence, *M*(*D*,*e*,*a*) is given by the following equation:

(3) $$M\left(D,\;e,\;a\right)=\;\mathrm{ERR}\left(D,e,\;a\right)\lambda_{I^{c}}\left(a\right)$$

where $\lambda_{I^{c}}$ represents the age‐ and sex‐dependent cancer incidence rate. The term *c* designates the site or type of cancer.

## 3 METHODS

This work aims to develop a software that provides the medical and technical staff of a nuclear medicine service (NMS) with a tool to establish reference levels in administered activity besides allowing for a more informed assessment of the risk of a procedure in relation to the benefit of a diagnosis or a therapeutic intervention. The software will allow knowing the doses involved in each procedure and respective reference levels in administered activity, allowing an analysis of the best procedure available for each pathology. The software developed in this study was used for a group of 2256 patients from National Cancer Institute (INCA), between 2017 and 2019. From this group of patients, a manual calculation of DRL, absorbed doses and risk for 100 patients were performed to validate the results obtained by the software. The methodology developed aims to reach the target audience who will be nuclear doctors, medical physicists, and NM specialists and meet the requirements of regulatory authorities. In general, the data obtained should reflect the local and later national level of practice, with the best possible quality.^8^

### 3.1 Diagnostic reference levels (DRLs)

The software, named NIREA, is in a web environment, improving software distribution. It was developed in PHP language, the function of which was to obtain diagnostic reference levels in activity (DRLA) for NM procedures by determining the third quartile of the exams performed.^8^ In this work, the influences of the installed technology (equipment in use) and the image quality produced for each procedure were not evaluated, as all patients are from the same service; it was considered that the doctors prescribing the activity to be administered and responsible for the final diagnosis present the same criteria for acceptance of the required quality.

The program also made it possible to obtain absorbed doses in organs based on some physical characteristics of the patients such as age, sex, and BMI to allow an analysis of the risk of each procedure. This tool allows the NM physician to review the activities prescribed to each patient and, thus, assess the risk–benefit involved.

Furthermore, gaps in the treatment prescription or variations in unjustified administrated activities for the same procedure can be identified, promoting protocol optimization.

### 3.2 Dose assessment

The absorbed doses in organs were estimated by the absorbed dose factor per administered activity (mGy/MBq) and the activity administered individually; the same process was applied for effective doses. The dose factors for each radiopharmaceutical were sought from the publications of ICRP 53103 (ICRP 53, 1987; ICRP 103, 2007).^9^, ^10^ However, these ICRP tables present the dose factors for fixed ages, namely, adults, 15, 10, 5 years, and 1 year. So, in the present study, an interpolation of the data was performed according to the radiopharmaceutical of interest. Charts listing the ages of children, teenagers, and adult references (ordered axis) with the absorbed dose conversion factors by unit of administered activity, mGy/MBq (abscissa axis), were generated. Likewise, the effective dose conversion factors were also related per unit of activity administered in mSv/MBq.

**FIGURE 1:**
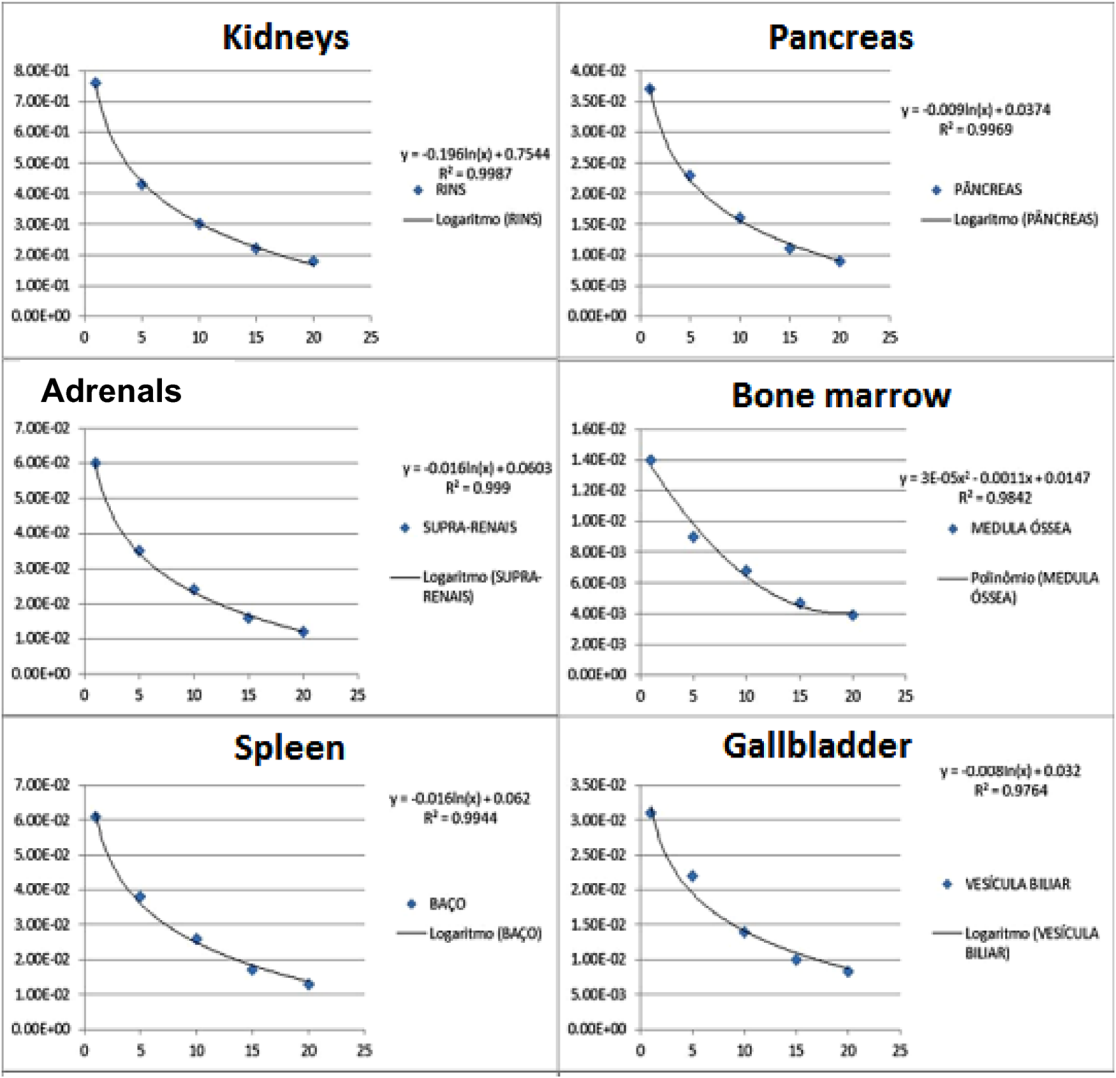
Interpolation graphs of age versus absorbed dose per unit of administered activity (mGy/MBq) to various organs for DMSA‐^99m^Tc (ICRP 53, 1988)

In Figure 1, some examples of the dose factor interpolations for the DMSA‐^99m^Tc protocol are shown.

The coefficient *R* ^2^ allows to identify how coherent the observed values are against a model; the closer to one (1), the better the quality of the fit. In the case of the graphs shown in Figure 1, it can be seen that, for all interpolations, the value of *R* approaches 1 from the first decimal place. Some points for organs such as medulla and gallbladder that do not were cut by the logarithmic curve, which indicates disparity in the remainder of the points that follow the mathematical behavior established by the logarithmic function. The logarithmic behavior of the graphs is because the younger the individual, the smaller the mass attributed to the organs, consequently increasing the absorbed dose, bearing in mind that this varies with the inverse of the organ mass. Another important factor for the age‐dependent dose factor is that the younger the individual, the more radiosensitive he will be; however, due to its high metabolism, the radiopharmaceutical will be excreted from the body more quickly than an adult patient.

### 3.3 Risk assessment

Models for specific cancer sites were applied to calculate the risks attributable to lifetime after medical exposure, based on an annual dose of reference organs/tissues and using statistical cancer incidence data from the Brazilian population.^11^ The risk assessment was performed by applying the mathematical models for calculating stochastic risk as proposed by BEIR VII Report 2.^7^ The transportation of the BEIR VII Committee's risk estimation models to a national population was carried out in two stages: (1) using the complete mortality tables for both sexes, published by the Brazilian Institute of Geography and Statistics (IBGE) to calculate the conditional probability *S*(*a*)/*S*(*e*); (2) using incidence and mortality rates for several types of cancer from INCA morbidity and mortality atlas.

Some critical organs were chosen, such as kidney, stomach, salivary glands, and bone marrow, in order to allow the estimation of the absorbed dose and subsequent calculation of the carcinogenic risk for each procedure. These were considered critical organs for possible deterministic and stochastic effects of radiation according to its biodistribution and accumulated activity.

By calculating the absorbed doses in the kidney, stomach, salivary glands, and bone marrow of each patient, the study used the dose values obtained to achieve two objectives: (1) estimate the risk of solid cancer in the first three sites; and (2) of leukemia due to the irradiation of the last one.

### 3.4 Pilot study to validate the software

The software was used in a cross‐sectional, retrospective observational study, analyzing a population of 2256 patients, between 2017 and 2019, undergoing diagnostic and therapy NM procedures at INCA in Brazil. This Institute is located in the state of Rio de Janeiro and belongs to the Public Unified Health System (SUS), that is, it serves the entire population without charge. It is the largest service provider by SUS in the state and fourth in the ranking of the largest hospitals in the country.

The database contains the following information: administered activity, sex, age, weight, and height. After classifying the patients by examination or treatment protocol, by sex and age range every 5 years, height and weight (BMI) data and the respective administered activities were included.

Among the different protocols performed in diagnosis, the following procedures were evaluated: static renal scintigraphy with DMSA, dynamic renal scintigraphy with DTPA, whole body scintigraphy with FDG, bone scintigraphy with MDP, myocardial scintigraphy with MIBI—resting protocol; myocardial scintigraphy with MIBI—stress protocol; parathyroid scintigraphy with MIBI—protocol.

This study was submitted and approved by the Research Ethics Committee of the INCA, Certificate of Presentation of Ethical Appreciation, Number 29217520.7.0000.5274.

## 4 RESULTS AND DISCUSSION

### 4.1 Software registration and operation

The NIREA software is available for free access at www.nirea.com.br. When accessing the website, the user has in the initial screen a brief description of the software and its functionality and has the requirement to do email registration. On the same screen, there are some buttons for contacts, support, registration, and login. To cover all regions of the country, the program provides a graphic resource that shows the DRLA by state of the federation, and that reproduces the information as it is fed by data from the NMS that adhere to the NIREA and share their data from procedures performed, thus generating a collaborative information network. As soon as the NMS is registered, its access is granted through a login and password, allowing a private and personalized statistical analysis, obtaining the local DRLA of the hospital or clinic itself. The software also provides a secondary platform for storing patient data, which is available for consultation whenever necessary.

NIREA has the function of generating reports on the NMS data so that the medical team has the DRLAs and their relationship with the values of absorbed dose and effective dose for each patient and procedure. These reports are intended to assist the NMS medical staff in standardizing the prescription of the activity to be administered by the different physicians; adjust patient‐administered activity for different ages, BMI, and gender groups; analyze the relationship between the effective dose and the absorbed dose as a function of the administered activity, allowing one more criterion to optimize the prescription and, consequently, the optimization of techniques, complying with regulatory requirements.

### 4.2 Diagnostic reference level in activity

In “dose map,” the user will select the desired options between the radionuclide type and exam. After this step, through the “search” function, a DRLA value will be made available on the map for each state of the federation. The user can position the cursor over the state of interest and by doing so an effective dose value will be provided for a specific exam. Given the differences between states, different values will be provided as the region varies. It is noteworthy that these data depend on the number of NMSs from each region registered on the map.

**FIGURE 2:**
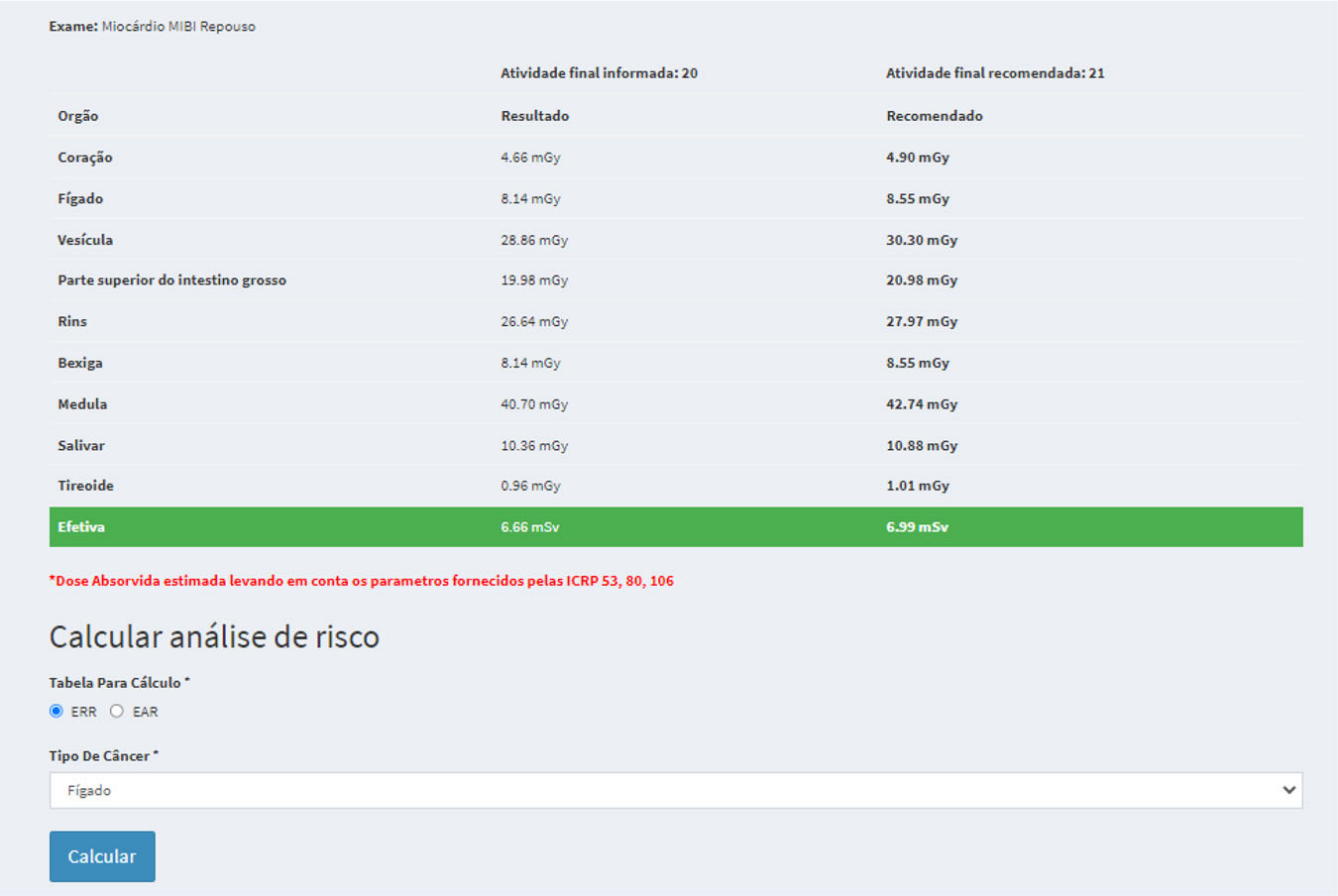
Dose map query

The second elementary function will be identified as “dose map query” where the consultation of absorbed doses in each organ can be performed by the user, as shown in Figure 2. This functional block allows obtaining absorbed dose information in critical organs for a given exam, depending on the administered activity reported by the user and in comparison with the DRLA, considering the characteristics of the individual that were entered in the buttons on the first line. The final activity to be provided by the user will be given by the difference between the prescribed activity and the residual activity in the syringe. By clicking on the “generate” button, the user will be redirected to a report page that will contain the information crossed between the software data and the entered data.

To exemplify the functionality of this software block, we selected a 40‐year‐old male patient who underwent an MIBI‐^99m^Tc cardiac myocardial examination with the resting protocol and administered activity of 740 MBq (20 mCi). With these data, the software makes available the absorbed doses estimated in critical organs based on the biokinetics of this radiopharmaceutical. Some of these organs are qualified for a risk analysis of radioinduced cancer using the absorbed dose as a parameter. In the case of the selected exam, the liver, bladder, thyroid, and kidneys are the organs that are enabled. Choosing the liver as an organ of interest by clicking on “risk calculation,” we are taken to a screen where we have graphical representations of the relative risk estimate, the mortality rate, and the LAR from the age of exposure.

**FIGURE 3:**
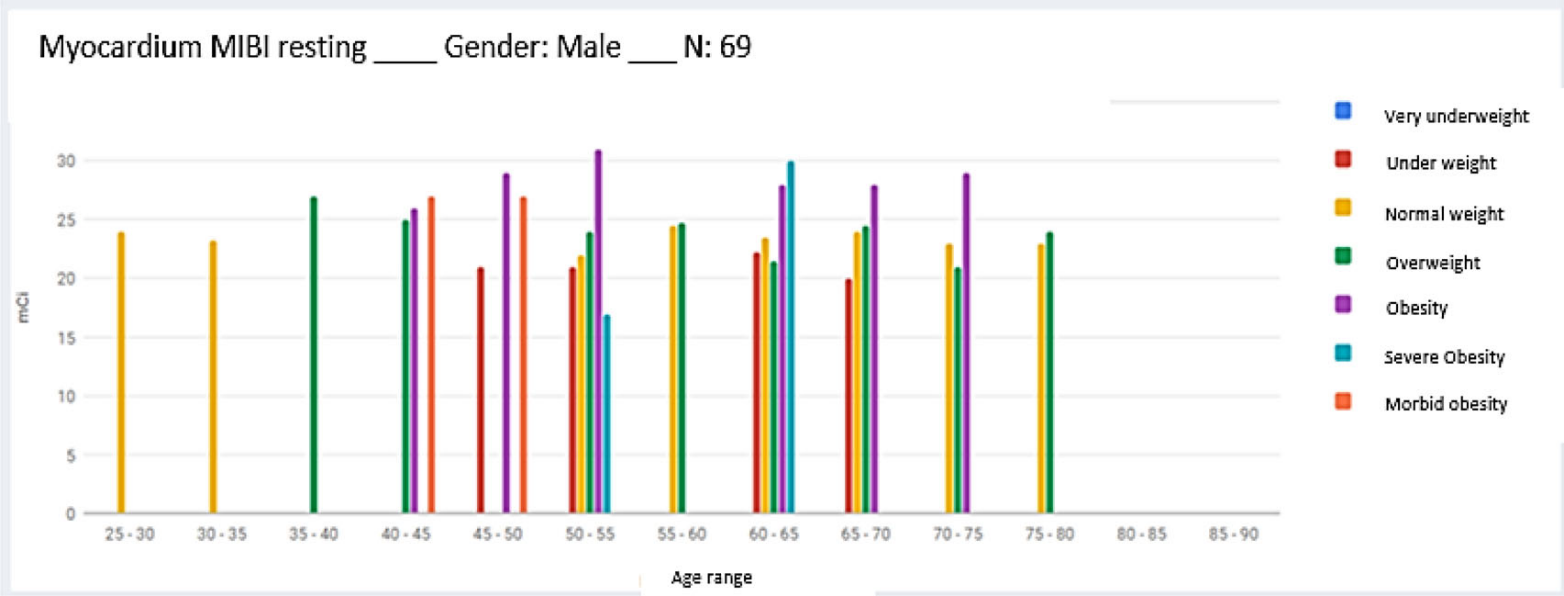
Example of the statistic report of diagnostic reference levels in activity (DRLA), applied to gender *male*, for procedure *Myocardium perfusion*, classified by body mass index (BMI) and age group

**FIGURE 4:**
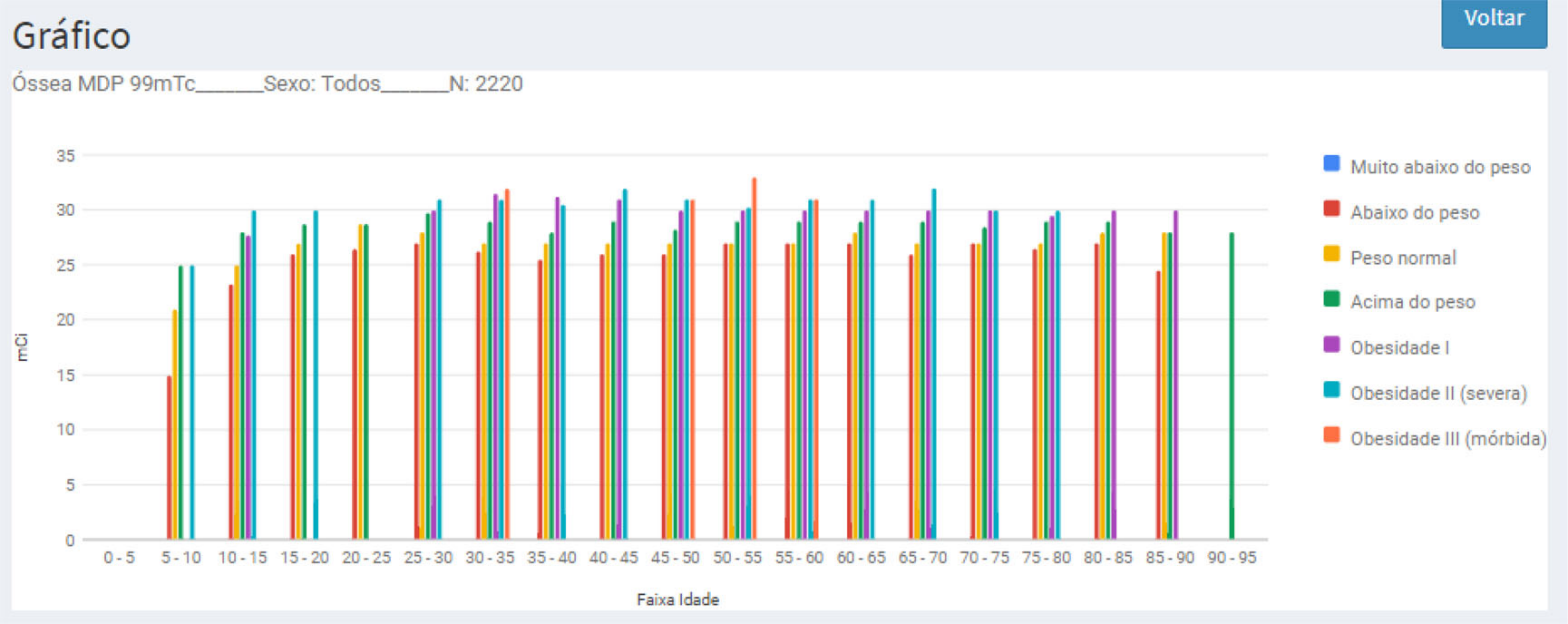
Example of the statistic report of diagnostic reference levels in activity (DRLA), applied to both gender, for procedure *MDP‐^99m^Tc* bone scintigraphy, classified by body mass index (BMI) and age group

The software also allows obtaining statistical reports that represent DRLA for each age group, segregated by BMI ranges, as shown in Figures 3 and 4.

This report allows a broader view of the activity distributions among the characteristics of patients in an NMS, facilitating a possible decision on where to focus the dose optimization procedures. Another feature is that the values of absorbed doses calculated for the activity administered to a specific patient are compared to the resulting ones based on statistics of activities performed at the national level, allowing for the assessment of differences. This comparison will guide the user's decision to maintain the prescribed activity or make the changes he deems necessary.

### 4.3 Risk analysis

For risk analysis, the user can select ERR or EAR models. Selecting the ERR button will provide the probability of cancer occurring from the exposure to which that patient was submitted. The EAR button, on the other hand, will provide the general probability of that individual having cancer considering different exposures throughout his life. After selecting the model for calculation, the user must select which organ he wants to do the risk analysis calculation.

**FIGURE 5:**
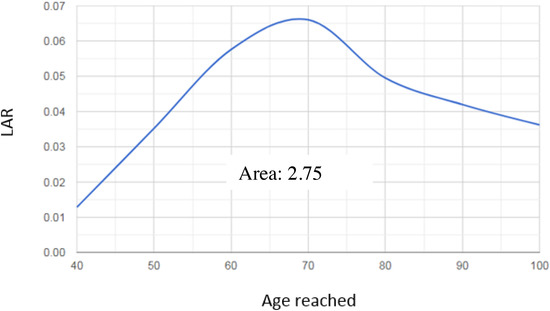
Lifetime attributable risk (LAR) plot for an absorbed dose of 8.14 mGy in the liver at 40‐year old as an age of exposure

In the result page, the user will find three graphics: ERR, *M*(*d*,*e*,*a*), and LAR. Following our example, the ERR graphic versus the age reached shows the quantitative estimate of risk due to the exposure suffered at 40 years of age and which may result in a radioinduced cancer in the liver over time. Local specific mortality given by *M*(*d*,*e*,*a*) expresses the product of the ERR with the mortality rate due to a specific cancer by sex and age of the Brazilian population. The LAR (Figure 5) is a function of *M*(*d*,*e*,*a*) in combination with the mortality rate of a general population. It results in an estimate of the risk associated with an exposure of interest during the useful life (rest of life) of an exposed population; technically, the lifetime risk is associated with exposure to dose *D*.

## 5 CONCLUSIONS

The software developed in this study made it possible an easy and objective methodology that allows obtaining DRLA for NM procedures based on patient characteristics. This tool also allows to perform a risk analysis of probability of a radioinduced cancer after the NM procedure, guided by the BEIR VII report through an LAR factor.

The absorbed dose calculation functionality, values were obtained for the critical organs studied in our example well below the thresholds for serious deterministic effects according to ICRP publication 118.^12^ Generally, the values established for the occurrence of deterministic effects are in the range of Gy, whereas the values obtained in diagnostic procedures are in mGy, indicating a low risk of occurrence of these effects. However, the risk of cancer induction turns out to be a stochastic effect and can be found in a low dose range.

This work showed that it is possible to transport a model developed to estimate the risk of carcinogenesis, LAR, for the Brazilian population using the national population's survival probabilities extracted from the mortality tables.

For the group of patients studied in this work, the absorbed doses remained in most cases at similar levels across the age spectrum, being generally as high in young patients as in adults. Given the greater susceptibility of younger patients to the stochastic effects of radiation, the findings suggest that a study of optimization of activities administered to young patients should be carried out in the service.

As a limitation of the study, we highlight that it is a cross‐sectional observational study, in which dosimetry was obtained by means of absorbed dose coefficients that were calculated based on the kinetics of a standard human model, the so‐called reference man, which may not satisfactorily reflect the biokinetics a specific patient. So, the proposed software should be considered a decision‐making tool, not an individual clinical dosimetry tool.

The NIREA software made it possible to perform calculations of the risk attributable to life for patients, according to the methodology of BEIR VII. Considering that the population of Brazil has genetic characteristics, probability of survival, and cancer incidence rates different from those observed in other countries, it is believed that the effort to obtain risk estimates adapted to our population was valid.

Further studies of the risk factors that influence basal cancer rates should be continued; thus, the improvement of risks for the Brazilian population can be obtained.

The developed program NIREA proved to be able to provide the estimated data of effective and absorbed doses in each NM procedure, for each patient, contributing to establish local, regional, and national DRLA, up to the present moment nonexistent in the national scenario.

A regional database was prepared containing the data of the NMS participating in this work for consultation by specialists in NM and for the national regulatory authorities, so that subsequently, with the increase in data acquisitions by different NMSs in the country, levels of availability can be made available in national practice. The developed and validated software is available on the website www.nirea.com.br for use by hospitals and clinics that have an NMS.

## AUTHOR CONTRIBUTIONS

Julio Cesar de Souza Ribeiro is the guarantor of the integrity of the entire study. J. D. S. Mendes contributed substantially to data acquisition during this study. All authors contributed to the study design, critical review of data analysis, and review of the manuscript and figures.
